# Potent induction of antibody-secreting B-cells by human dermal-derived CD14+ dendritic cells triggered by dual toll-like receptor ligation

**DOI:** 10.1186/1742-4690-9-S2-P12

**Published:** 2012-09-13

**Authors:** K Matthews, NP Chung, PJ Klasse, JP Moore, RW Sanders

**Affiliations:** 1 Weill Cornell Medical College, New York, NY, USA; 2 Academic Medical Center, Amsterdam, Netherlands

## Background

A goal of HIV-1 vaccine development is to induce broadly neutralizing antibodies. However, the Env complex is poorly immunogenic and requires potent adjuvants. Given the pivotal role of TLRs and DCs in initiating and tuning adaptive immune responses, TLR agonists are attractive adjuvants. CD14+ dermal DCs (CD14+ DDCs) have a natural capacity to stimulate naïve B-cells, so targeting these cells with TLR ligands is a rational approach to inducing humoral responses.

## Methods

Migratory cells were collected after culturing skin for 24 h. CD14+ DDCs were purified using CD14 magnetic beads, stimulated with TLR ligand(s) and analyzed for cytokine expression (ELISA/qPCR) and phenotype after 48 h. Naïve B-cells were stimulated with TLR ligand(s) plus CD40L and IL-2, either alone or in the presence of CD14+ DDCs, and analyzed for proliferation, phenotype and IgG/IgA secretion. TLR-ligand stimulated DDCs were incubated with allogeneic naive CD4+ T-cells for 6 days before T-cell derived cytokines were quantified.

## Results

CD14+ DDCs express mRNA for TLRs 1−9, but respond differentially to single or paired TLR ligands. Compared to single ligands, some combinations were particularly effective, increasing the expression of B-cell stimulatory cytokines and maturation of the DDCs. These combinations were R-848 plus Poly(I:C); R-848 plus LPS; Pam3CSK4 plus Poly(I:C); LPS plus Poly(I:C). Selected TLR agonist pairs (R-848 plus either LPS or Poly(I:C)) were superior to individual agents at boosting the capacity of CD14+ DDCs to induce naïve B-cells to proliferate and differentiate into CD27+CD38+ B-cells that secrete high levels of IgG and IgA. These selected TLR ligand combinations also induced CD14+ DDCs to promote differentiation of Th1, but not Th2, Th17 or TFH cells.

## Conclusion

Two TLR ligand combinations potently activate CD14+ DDCs to have enhanced B-cell stimulatory capacity, and could be used to improve humoral immune responses to HIV-1 Env.

